# Plain Language Summarization of Environmental Health Research Using Generative AI: Community-Engaged Qualitative Study

**DOI:** 10.2196/87118

**Published:** 2026-05-13

**Authors:** Katherine Wade, Lauren B Anderson, Evelyn M Medley, Ted Smith

**Affiliations:** 1 Christina Lee Brown Envirome Institute School of Medicine University of Louisville Louisville, KY United States; 2 Department of Epidemiology and Population Health School of Public Health and Information Sciences University of Louisville Louisville, KY United States; 3 Superfund Research Center University of Louisville Louisville, KY United States; 4 Department of Urban and Public Affairs College of Arts and Sciences University of Louisville Louisville, KY United States; 5 Berea College Berea, KY United States

**Keywords:** generative artificial intelligence, generative AI, large language models, plain language summaries, environmental health, community-based participatory research, environmental justice, research translation, health communication

## Abstract

**Background:**

Plain language summaries (PLSs) are increasingly required in environmental health publications to improve public accessibility. Generative artificial intelligence (AI) systems such as large language models can automatically produce such summaries; however, automated summaries often overlook local context and cultural relevance—limitations that are critical in environmental health research, where affected communities face disproportionate exposure and health risks.

**Objective:**

This study aimed to develop and refine community-informed prompts for generating PLSs of environmental health research using generative AI.

**Methods:**

We conducted a community-engaged qualitative study in Louisville, Kentucky, involving workshops with 97 participants from 4 stakeholder groups: summer interns at a local social justice nonprofit organization, participants in a youth development program, participants in a faith-based community organization, and members of a community advisory board focused on environmental justice. Participants reviewed PLSs generated using 3 different prompt styles in GPT-4o (ChatGPT, OpenAI). Feedback was collected through structured discussions and facilitator notes. Data were analyzed using thematic analysis informed by knowledge translation frameworks to identify preferred structural, linguistic, and contextual features of the summaries.

**Results:**

Participants consistently preferred summaries between 300 and 400 words, written at a sixth- to eighth-grade reading level. Key priorities included presenting definitions before findings, using headings and bullet points to improve readability, and clearly explaining real-world and environmental justice implications. Narrative summaries without structure were viewed as overly long and difficult to interpret, while purely bullet-based formats were considered too simplified. Feedback from youth participants emphasized clarity and practical relevance, while faith-based participants highlighted the importance of trust and contextual framing. These insights informed the development of a community-refined prompt that included definitions, key findings, an introduction, and a concluding section on community implications.

**Conclusions:**

Community-engaged prompt development may improve the relevance and interpretability of AI-generated PLSs of environmental health research. Incorporating stakeholder perspectives into prompt design offers a replicable strategy for improving research translation and ensuring AI-generated summaries reflect the informational needs of affected communities.

## Introduction

### Background

Plain language summaries (PLSs) aim to bridge the gap between scientific communication and public understanding [1]. In environmental health research, where technical language often obscures public health implications, PLSs are particularly critical [2]. Although publishers, platforms, and even search engines now provide standardized artificial intelligence (AI)–generated lay summary functions, these “precanned” summaries frequently overlook local context, cultural framing, and specific community concerns [1,2]. Studies examining automated summarization in scientific publishing note that although such tools improve accessibility, they often fail to capture place-based relevance, omit social factors, and lack sensitivity to community-level variation in risk perception [1,3]. For instance, an automated summary of a study on air pollution and heart disease might highlight general health effects but omit that the research was conducted in a predominantly Black neighborhood disproportionately exposed to industrial emissions due to historical redlining—a context that is potentially vital for community understanding and action.

The growth of generative AI has accelerated opportunities to create PLSs but has also raised questions of accuracy, inclusivity, and transparency [4]. Community-informed approaches, rooted in established models of engagement [5,6], offer a pathway to developing prompts that are better aligned with the needs of nonexpert but highly invested audiences.

### Environmental Health Literature Lay Summarization

Many journals in environmental health and biomedical sciences now require or encourage PLSs (Textbox 1).

**Examples of lay summary formats in environmental health and biomedical journals.**

Several publishers and organizations have adopted distinct formats for presenting plain language summaries:

- Cochrane provides detailed templates requiring structured sections such as “What did we find?” and “What are the limitations?” [7].
- eLife has pioneered plain language “digests” as an integral component of their publications [8].
- Journals in PLOS contain concise “Author Summary” sections targeted at lay readers [1].

Although it is not possible to know how many of these summaries are created by the author without AI assistance, the notion that research publications should include accessible summaries has become a convention in scholarly publications. Additionally, some publishers such as Wiley and Elsevier have incorporated AI-generated summaries into their online presentation of papers [9]. Table 1 compares lay summary formats currently used across journals and AI platforms.

**Table 1:** Comparison of lay summary prompts across journals and platforms.

Source	Format or prompt style	Primary audience	Notable features
Cochrane	Structured template	Patients and policymakers	Sections such as “What did we do?” “What did we find?” “What are the limitations?”
eLife	Narrative “digest”	General public	Short, story-style explanation written alongside the article
PLOS journals	Author summary	Nonspecialist readers	Concise summary (approximately 250 words) describing the significance of the research
Wiley	Automated research summary	General and academic audiences	Machine learning–generated article highlights presented on article web pages
*Environmental Health Perspectives*	Short plain language summary or highlight box	Policy and practice audiences	Journalistic-style summaries emphasizing relevance to public health
Google Gemini (search integration)	Artificial intelligence–generated abstract simplifications	General users	Automated summaries generated dynamically within search results

Beyond these features in journal publication, generative AI has become an automatic feature when these articles are discovered in Google searches with the recently integrated Gemini (version 3.1; Google LLC)-generated summaries, and most large language models now generate automatic “key findings” or “about this study” snippets when academic articles are searched, functioning as default lay summaries. Although these features democratize access, they risk missing the types of information needs that can be met through an iterative, context-sensitive cocreation process that community engagement can enable.

The substance and style of an AI-generated summary are directly mediated by the nature of the prompt. Even small adjustments in phrasing—such as specifying audience, tone, or structure—can produce markedly different outputs from the same model [10]. Prompt wording determines not only what information is emphasized but also how it is framed, influencing readability, inclusivity, and factual precision [4]. This sensitivity underscores that prompt design is not a neutral technical process but a critical determinant of research translation. In the context of environmental health, where issues of justice and accessibility are paramount, the ability to guide AI outputs toward equity-oriented narratives depends on the intentionality of the prompts themselves [2]. This study aimed to explore a community-engaged approach to developing prompts for generative AI systems to produce PLSs of environmental health research that are accessible, contextually relevant, and meaningful to affected communities [5,6]. Our specific aim was to codevelop and refine prompts for GPT-4o (ChatGPT, OpenAI) through direct engagement with diverse community stakeholders in Louisville, Kentucky.

## Methods

### Setting and Participants

Engagement workshops were conducted in partnership with 3 community-based groups that represent populations disproportionately impacted by environmental exposures and health disparities. A total of 97 participants took part in the workshops, including youth participants from a mentoring program (n=81, 83.5%), members of a community advisory board focused on environmental health (n=10, 10.3%), participants from a faith-based environmental justice initiative (n=6, 6.2%), and student interns at a social justice nonprofit organization (n=5, 5.2%). Prompt development followed an iterative refinement process across multiple engagement sessions. Early sessions (June-July 2024) focused on comparing alternative prompt structures (eg, narrative vs bullet format and varying reading levels), while later sessions (October 2024) evaluated refined prompts incorporating structured sections and definitions.

Detailed demographic data such as age, gender, race or ethnicity, or education level were not collected during the workshops because the activities were conducted as community engagement sessions rather than formal participant surveys.

### Workshop Procedures

Workshops were cofacilitated by environmental health academic researchers and community liaisons. Each session began with an orientation to the purpose of PLSs, examples of generative AI, and ethical considerations around AI use. Participants were provided with summaries generated using GPT-4o of 3 environmental health journal papers [11-13]. Papers were selected based on their authorship by readily accessible Superfund researchers and their relevance as current publications.

All PLSs were reviewed by study authors for accuracy, completeness, and readability. Although interrater reliability was not assessed (because the authors reviewed their own papers), the review process ensured scientific fidelity. Facilitators used structured discussion guides and note-taking templates to ensure consistency across sessions. Feedback was collected through a combination of small-group discussions, full-group debriefs, and facilitator-recorded observations. In most sessions, feedback was documented in real time by at least 1 team member, and in some sessions, multiple facilitators contributed to note-taking to ensure completeness.

### PLS Prompts

Participants evaluated PLSs generated using 3 different prompt structures (refer to Textbox 2 for details).

**Plain language summary prompt structures evaluated in the study.**

The following prompts represent the initial and refined instructions used to generate plain language summaries for participant evaluation:

- Prompt A (initial draft): “Summarize the research paper in 600 words at an eighth-grade reading level. The entire summary should be in a narrative format.”
- Prompt B (initial draft): “Summarize the research paper in 300 words at a sixth-grade reading level. The entire summary should be in a bullet format.”
- Prompt C (refined draft): “Summarize the provided text using simple and clear language. Start with key findings followed by translations for technical terms. Organize the summary using headings for ease of reading. Include the following:
- Complete title and all authors
- Key findings: list the main results or conclusions
- Definitions: translate any technical terms used in the summary
- Introduction: provide a brief overview of the topic
- Main content: discuss the significant points, organized with headers as needed
- Conclusion: summarize the overall findings and their implications

Ensure the summary is suitable for a nonexpert audience. Do not include the Methods or Results sections in the summary.”

Participants reviewed the summaries created by each of these prompts individually and in small groups, then discussed strengths, weaknesses, and preferred features. Only prompt C specified the use of section headings and structured subsections; prompts A and B generated narrative and bullet-based format summaries without headings. Facilitators recorded real-time feedback using structured templates and open-ended note-taking (refer to Multimedia Appendices 1 and 2 for the PLS protocol and complete examples of prompts and generated PLSs).

### Analytic Approach

Feedback from workshops was documented through structured templates and detailed facilitator notes recorded during and immediately after each session. Across all engagement activities (June-October 2024), notes captured both group-level discussion and specific participant comments regarding clarity, structure, tone, and perceived usefulness of each PLS. The designated note-taker documented the principal elements of participants’ contributions and reviewed them with the group prior to the conclusion of the session to ensure accuracy. We conducted a thematic analysis drawing on knowledge translation frameworks [9] and empirical research on plain language summarization [1], using an iterative, multistage approach. First, all notes were compiled across sessions and reviewed to identify recurring patterns in participant feedback. Members of the research team performed initial open coding to label discrete concepts (eg, “need for simpler language,” “preference for definitions,” “desire for personal relevance,” and “confusion with technical terms”). These codes were informed both by emergent participant feedback and by prior literature on plain language communication and knowledge translation.

Second, codes were grouped into higher-order thematic categories through an inductive-deductive process. Themes were refined through comparison across multiple community groups (youth participants, community advisory board members, and faith-based participants) to identify consistent versus group-specific priorities. For example, youth participants emphasized personal relevance and visual elements, while advisory board participants more frequently commented on clarity, completeness, and scientific accuracy.

Third, themes were organized into three overarching domains: (1) structural (eg, use of headings, placement of key findings, and inclusion of definitions); (2) linguistic (eg, reading level, jargon reduction, and clarity of explanations); and (3) contextual (eg, relevance to lived experience, environmental justice framing, and implications for personal or community health).

To enhance analytic rigor, findings were triangulated across multiple sessions conducted at different times and with distinct participant groups (June, July, and October 2024). Consistency of feedback across sessions, such as repeated calls for simpler language, clearer definitions, and emphasis on personal relevance, was used to confirm theme stability. Finally, themes were applied to iteratively refine the prompt design. Feedback directly informed the development of the final structured prompt (prompt C), which incorporated prioritized features, including key findings at the beginning, explicit definitions of technical terms, and organized headings for readability. A summary of thematic guidelines and sample feedback is provided in Multimedia Appendix 3.

### Ethical Considerations

The project was reviewed by the University of Louisville Institutional Review Board (IRB) and was deemed inapplicable for the Common Rule definition of human subjects research because the activities were classified as program evaluation and quality improvement rather than research involving identifiable human subjects. The IRB issued a formal determination of non–human subjects research (IRB 25.0911; reference 813263) on February 2, 2026.

Workshop participants engaged in group discussions evaluating PLSs generated using generative AI tools. No personal identifiers were collected during the workshops, and all feedback was documented through facilitator notes in deidentified form. Because the activity was determined to be non–human subjects research, formal informed consent procedures were not required. Participants were informed about the purpose of the workshop activities and participated voluntarily. Participants did not receive compensation for participation, although food and beverages were provided.

All project activities followed University of Louisville policies regarding data security and privacy. Feedback collected during workshops was stored on institutionally approved systems and analyzed only in an aggregated, deidentified form. No participant-level data are reported in this manuscript. Youth participants were involved through community partner programs that follow institutional youth protection policies and guidelines.

## Results

### Participant Characteristics

A total of 97 individuals participated in 3 community engagement workshops conducted in Louisville, Kentucky. Participants represented 3 community partner groups: a youth development and mentoring program (n=81, 83.5%), a community advisory board focused on environmental health and research engagement (n=10, 10.3%), and a faith-based organization involved in environmental justice initiatives (n=6, 6.2%). The youth participants were primarily high school students, while the advisory board and faith-based groups consisted of adult community members with varying levels of experience interacting with environmental health research.

The large proportion of youth participants provided insight into how younger audiences interpret scientific information presented in simplified formats. Participants across all groups were familiar with environmental concerns in their communities, including air pollution and industrial exposures, and were therefore well-positioned to evaluate whether the summaries communicated research findings in ways that were understandable and relevant.

### Evaluation of Prompt Formats

Participants reviewed PLSs generated using 3 different prompt structures. These prompts varied in length, format, and the level of structural guidance provided to the generative AI system.

Prompt A generated summaries of approximately 600 words written in a narrative format at an eighth-grade reading level. Participants in the community advisory board workshop generally perceived this format as overly long and dense. Several participants commented that the summaries felt similar to reading a condensed research paper rather than a plain language explanation. Although the summaries contained detailed explanations of study findings, participants noted that key messages were sometimes difficult to identify quickly.

Prompt B produced summaries of approximately 300 words written in a bullet-point format at a sixth-grade reading level. Participants reported that this structure made the summaries easier to skim and understand. Youth participants in particular appreciated the shorter length and visual organization of the bullet points. However, both youth and adult participants noted that this format sometimes oversimplified the research findings and lacked sufficient explanation of key concepts or context. Participants also reported that the bullet-point format occasionally made the summaries feel fragmented or disconnected from the broader purpose of the study.

Prompt C incorporated a structured format with headings, definitions of technical terms, and clearly separated sections describing key findings and implications. Participants across all workshops responded more favorably to this structure. The use of headings and defined sections allowed readers to navigate the summary more easily and locate the most important information. Participants also expressed appreciation for the inclusion of definitions that explained technical terminology before presenting the research findings.

### Emergent Themes From Community Feedback

Across the 3 workshops, several consistent themes emerged regarding preferred characteristics of PLSs generated using AI tools. These themes related to the structure, language, and contextual framing of the summaries.

First, participants emphasized the importance of presenting definitions of technical terms before introducing the research findings. Participants explained that encountering unfamiliar terminology early in a summary could make the content difficult to follow. Providing definitions at the beginning helped establish a clearer understanding of the scientific concepts discussed later in the summary.

Second, participants consistently expressed a preference for concise summaries. Many participants indicated that summaries of between approximately 300 and 400 words struck an appropriate balance between readability and informational depth. Summaries longer than this were perceived as burdensome to read, while shorter summaries sometimes omitted important context.

Third, participants emphasized the importance of clearly explaining why the research matters. In particular, youth participants frequently asked how the findings related to their own communities or daily lives. Participants indicated that summaries should explicitly describe potential health implications and identify populations that may be most affected by the environmental exposures being studied.

Finally, participants highlighted the importance of contextual framing related to environmental justice. Adult participants in the advisory board and faith-based workshops noted that environmental health research often occurs in communities disproportionately exposed to pollution. They recommended that summaries explicitly acknowledge these broader social and environmental contexts when describing study findings.

### Development of the Community-Refined Prompt

The themes identified through workshop discussions informed the development of a revised prompt designed to generate more accessible and contextually relevant summaries. The final prompt incorporated several features that participants consistently preferred. These included presenting definitions of technical terms at the beginning of the summary, organizing information into clearly labeled sections, and highlighting key findings using concise bullet points.

In addition, the revised prompt instructed the AI system to include a short, narrative introduction and conclusion describing the broader implications of the research. Participants emphasized that these sections helped connect the scientific findings to real-world concerns, including environmental health risks and community-level impacts.

Therefore, the final prompt combined both structured and narrative elements to improve readability while preserving important contextual information. When applied to environmental health research papers, this prompt produced summaries that participants described as clearer, more trustworthy, and more relevant to community audiences. The final prompt was not subjected to systematic re-evaluation by participants. Subsequent research efforts are directed toward assessing the resulting summaries with a diverse range of stakeholders within the community. Table 2 summarizes participant feedback on each prompt structure and the features that informed the final community-refined prompt.

These findings echo the observation by Anderson et al [2] that prompts emphasizing readability and real-world implications were rated more effective by study authors. Together, these results suggest that audience-sensitive prompt design—guided by both researchers and communities—yields better outcomes than generic summarization.

**Table 2:** Summary of participant feedback on plain language summary prompt formats.

Prompt	Prompt description	Community group	Key feedback
Prompt A	Summarize the research paper in 600 words at an eighth-grade reading level using a narrative format	Community advisory board	Considered too long and repetitive; participants described it as reading like a condensed research report rather than a summary
Prompt B	Summarize the research paper in 300 words at a sixth-grade reading level using bullet points	Youth development program	Easier to skim and understand but sometimes oversimplified findings and lacked context
Prompt C	Use a structured format, including a title, definitions, key findings, an introduction, main content, and a conclusion	Community advisory board and faith-based community participants	Most preferred format; headings and definitions improved readability and clarity
Final community-refined prompt	Summarize the research paper using simple, community-accessible language (sixth- to eighth-grade reading level). Include a citation, definitions, key findings, an introduction, and a conclusion. Highlight implications for affected communities and environmental justice	All groups combined	Viewed as clear, trustworthy, and relevant; participants valued the presentation of definitions first and contextual explanations of community impact

## Discussion

### Principal Findings

This study explored how community-engaged feedback can inform the development of prompts for generative AI systems used to produce PLSs of environmental health research. Across 3 community workshops involving youth participants, community advisory board members, and faith-based stakeholders, participants evaluated AI-generated summaries created using different prompt structures. The findings demonstrate that the prompt structure is not a neutral technical input; rather, it shapes how scientific findings are organized, what contextual information is emphasized, and how easily community audiences can identify the relevance of environmental health research.

Participants consistently preferred summaries that were concise, clearly structured, and written at approximately a sixth- to eighth-grade reading level. Narrative summaries generated using a longer prompt format were often perceived as overly dense and difficult to navigate, even when they contained detailed explanations of the research. In contrast, shorter bullet-based summaries were easier to read but were sometimes viewed as overly simplified or lacking sufficient context. The most widely preferred format combined structured headings, definitions of key terms, and concise descriptions of findings with brief narrative explanations of the study’s relevance.

Across all workshops, participants emphasized 4 priorities for effective AI-generated summaries: presenting definitions of technical terms before introducing findings, maintaining a concise length of approximately 300 to 400 words, clearly explaining why the research matters, and situating the findings within broader environmental justice contexts. These insights informed the development of a community-refined prompt that incorporated both structured and narrative elements designed to improve readability and contextual relevance.

Together, these findings suggest that prompt design plays a critical role in shaping how generative AI systems translate scientific information for nonspecialist audiences. Therefore, incorporating community perspectives into prompt development may improve the accessibility and usefulness of AI-generated summaries for audiences most affected by environmental health research.

### Comparison With Existing Literature

The findings of this study are consistent with prior literature emphasizing the importance of PLSs in improving access to scientific information among nonspecialist audiences. Systematic and scoping reviews have found that PLSs are intended to make research more understandable, accessible, and usable but have also reported substantial variability in summary format, quality, readability, and guidance for authors [1,14]. Our findings support this literature by showing that community audiences value core features commonly recommended for plain language communications including reduced jargon, clear structure, concise wording, and explicit explanation of technical terms. Similarly, emerging research on generative AI in environmental health communication suggests that AI tools may assist in research translation but require careful prompt design to ensure that outputs remain accurate, readable, and relevant to intended audiences [2].

At the same time, our findings extend prior PLS research in several ways. Much of the existing literature focuses on author guidance, publisher requirements, readability metrics, or expert evaluation of summaries [1,14,15]. In contrast, this study examined how intended community audiences evaluate AI-generated summaries and how their feedback can be used to refine the prompts that produce those summaries. This distinction is important because a summary that satisfies general readability criteria may not necessarily meet the information needs of a specific community. Participants in this study did not evaluate summaries only based on the reading level; they also assessed whether the summaries explained why the research mattered, whether key terms were introduced in a logical order, and whether the findings were connected to environmental health concerns relevant to community experience.

Recent literature has also noted that PLSs vary in their adherence to health literacy principles and that inconsistent guidance may limit their usefulness for public audiences [14,15]. Our findings provide a practical response to this challenge by identifying specific prompt elements that can operationalize health literacy principles in AI-generated summaries. For example, prompts can instruct the model to define technical terms before presenting findings, limit summary length, use headings that guide readers through the content, and include a brief “why this matters” section. These prompt features translate general recommendations for plain language communication into reproducible instructions that can be applied across environmental health studies.

Our results also complicate the assumption that shorter or simpler summaries are always preferable. Participants appreciated the readability of bullet-based summaries, but they also noted that overly brief summaries could omit important context. This finding aligns with recent cautions in AI summarization research that simplification can introduce tradeoffs, including loss of nuance, overgeneralization, or reduced factual completeness [16,17]. In this study, participants preferred a hybrid format that balanced brevity with sufficient explanation. This suggests that readability metrics alone are insufficient for evaluating AI-generated PLSs. A summary may score well on reading-level measures while still failing to explain the significance of the findings or their implications for affected communities.

The findings further reinforce the importance of human review and audience testing in AI-mediated research translation. Recent studies of AI-generated lay summaries have emphasized the need for editorial oversight, domain expertise, and user evaluation to ensure that summaries remain accurate, understandable, and appropriate for intended audiences [16,18,19]. Our findings support these recommendations and suggest that community review may be especially important in environmental health contexts, where scientific findings often intersect with lived experience and local concerns about exposure and risk.

### Community-Engaged Environmental Health Translation

The results of this study also align with broader frameworks for community-engaged research, environmental health literacy, and knowledge translation. Environmental health literacy emphasizes not only the ability to understand environmental health information, but also the capacity to use that information in ways that support awareness, decision-making, and action [20]. Similarly, community-engaged environmental health research emphasizes bidirectional communication between researchers and communities, including the translation of scientific findings into formats that are culturally appropriate, relevant, and responsive to community priorities [21].

Our findings illustrate why this bidirectional approach is important for AI-generated research summaries. Participants did not simply ask for summaries to be shorter or easier to read. They asked for summaries that clarified technical language, explained the real-world meaning of findings, and connected research results to environmental justice issues. This emphasis is particularly relevant in environmental health research, where exposure risks, pollution burdens, and health outcomes are often shaped by structural and social dynamics. A generic AI-generated summary may accurately describe a study’s methods and results while still failing to communicate why the findings matter to communities experiencing disproportionate environmental burdens.

Recent environmental justice guidance has also emphasized the need for accessible public-facing communication formats, including PLSs, visual tools, and other dissemination products that make scientific information more usable for affected communities [22]. Our findings provide empirical support for these recommendations by identifying features that community stakeholders perceived as most useful in AI-generated environmental health summaries. In particular, participants valued summaries that included definitions, clear organization, concise findings, and explicit discussion of community relevance. These features may help bridge the gap between scientific dissemination and community-centered environmental health communication.

Therefore, this study positions prompt engineering as more than a technical process. In the context of environmental health research translation, prompt design functions as a communication practice that determines how scientific knowledge is framed for public audiences. Decisions about whether to include definitions, how to describe uncertainty, whether to mention environmental justice, and how to explain real-world relevance all shape the meaning of the summary. Community-engaged prompt development offers a mechanism for making these decisions more transparent and responsive to the needs of intended audiences.

### Implications for AI-Generated Research Translation

These findings have practical implications for researchers, journals, and technology platforms seeking to integrate generative AI into research communication workflows. First, the results highlight the importance of explicitly specifying audience characteristics in prompt design. Participants strongly preferred summaries written at reading levels consistent with general public literacy levels and organized in ways that supported rapid comprehension. Prompts that simply ask an AI system to “summarize this article in plain language” may be insufficient because they do not specify audience, structure, length, or terminology.

Second, participants emphasized the importance of contextualizing scientific findings within real-world environmental health concerns. In environmental health research, issues such as pollution exposure, structural inequities, and environmental justice are central to understanding the significance of scientific findings. Generic AI-generated summaries may overlook these contextual factors unless prompts explicitly instruct the model to address them.

Third, the study suggests that community engagement can play a meaningful role in shaping how generative AI tools are used in research dissemination. Rather than relying solely on standardized prompts developed by publishers or software developers, researchers may benefit from incorporating feedback from community stakeholders who represent intended audiences for research findings. This participatory approach aligns with broader frameworks for community-engaged research and knowledge translation, which emphasize collaborative processes for interpreting and communicating scientific evidence [20,21,23].

An important challenge in AI-mediated summarization is that the priorities researchers seek to communicate may not always fully align with the issues that communities view as most relevant. Researchers may emphasize methodological rigor, statistical findings, or theoretical implications, whereas community audiences may be more interested in practical health implications, environmental justice concerns, or actions that could reduce exposure risks. Generative AI tools provide a potential mechanism for balancing these perspectives because prompts can be structured to incorporate both researcher-defined priorities and community-informed framing. For example, prompts may require summaries to include core scientific findings identified by the authors while also incorporating sections that explicitly address community relevance or real-world implications. In this way, AI-mediated summarization does not replace researcher interpretation but instead enables multiple layers of translation that reflect both scientific priorities and community information needs. Although journals often rely on standardized summary formats to maintain consistency and scalability, generative AI systems may allow authors to generate both standardized summaries and audience-adapted versions using alternative prompts tailored to specific audiences or communities.

### Limitations

Several limitations should be considered when interpreting these findings. First, the workshops were conducted with community groups located in a single metropolitan area, which may limit the generalizability of the results to other geographic or cultural contexts. Second, most participants were youths involved in a mentoring program. Although this group provided valuable insight into how younger audiences interpret scientific summaries, their preferences may differ from those of other populations, including older adults or individuals with professional experience in environmental health fields. In addition, individual-level demographic information was not collected during the workshops, which limits the ability to analyze how participant characteristics such as age, race, or educational background may have influenced preferences for summary formats.

Third, the study relied on qualitative feedback collected through workshop discussions and facilitator notes. Although this approach allowed participants to provide rich insights into how they interpreted different summaries, the study did not include quantitative measures of readability, comprehension, or trustworthiness. Consequently, the findings reflect participant perceptions rather than objective performance comparisons between prompt structures.

Finally, the AI-generated summaries evaluated in this study were produced using a specific generative AI system and a limited set of environmental health articles. Results may vary when using different models, prompt configurations, or research topics.

### Future Directions

Future research should examine how community-informed prompt design influences the effectiveness of AI-generated summaries across different populations and communication settings through more rigorous study designs. Quantitative studies measuring readability, comprehension, and trust in AI-generated summaries could help evaluate whether community-informed prompts improve audience understanding compared with generic summarization approaches.

In addition, future work could explore how AI-generated summaries might be adapted for multilingual or multimodal communication formats. Several workshop participants noted that alternative formats such as infographics, short videos, or audio summaries may further improve accessibility for individuals with varying literacy levels or learning preferences. At the same time, ensuring that simplified or AI-generated summaries do not omit important context or lead to misinterpretation will be an important challenge. Therefore, future implementations may benefit from transparent prompt design and continued researcher or editorial oversight to ensure that summaries accurately convey key findings and limitations.

Finally, the integration of community-informed prompts into journal publication workflows represents an important area for future exploration. Journals and publishers increasingly require PLSs as part of the publication process, and generative AI tools may assist authors in producing these summaries efficiently. Incorporating community perspectives into prompt design could help ensure that these summaries communicate research findings in ways that are both scientifically accurate and meaningful to affected communities.

### Conclusions

This study adds to the literature on PLSs, AI-generated lay communication, and community-engaged environmental health translation by demonstrating that community feedback can directly inform prompt design for generative AI systems. The rapid emergence of generative AI systems has created new opportunities to automate aspects of this process, but the effectiveness of AI-generated summaries depends heavily on how prompts are designed. As the discourse evolves, ongoing research on prompt crafting and its ethical implications within environmental contexts remains imperative for achieving just and equitable outcomes in AI-generated information. This study demonstrates that engaging community stakeholders in prompt development can help identify structural and contextual features that make AI-generated summaries more understandable, relevant, and trustworthy for nonspecialist audiences. By incorporating community feedback into prompt engineering, researchers can better align AI-generated summaries with the informational needs of populations most affected by environmental health risks. As generative AI becomes more widely integrated into research dissemination, community-informed approaches to prompt design may provide an important pathway for improving the translation of scientific knowledge into accessible and actionable information.

## Appendix

Multimedia Appendix 1 Plain language summary protocol.

Multimedia Appendix 2 Complete plain language summary examples from focus groups.

Multimedia Appendix 3 Feedback categorization for summaries.

## Abbreviations

AI: artificial intelligence
IRB: institutional review board
PLS: plain language summary

## Data Availability

The qualitative data generated during this study consist of facilitator notes and thematic coding summaries and are not publicly available due to the potential identifiability of participants. Deidentified excerpts may be available from the corresponding author upon reasonable request.
