# Supplementary material for: Plain Language Summarization of Environmental Health Research Using Generative AI: Community-Engaged Qualitative Study
Source: J Med Internet Res. 2026 May 13;28:e87118. doi: 10.2196/87118 (PMC13216757; doi:10.2196/87118)
Supplement: Multimedia Appendix 1 [file jmir_v28i1e87118_app1.docx]

**Appendix 1: Plain Language Summary Protocol**

**Plain Language Summary Protocol**

1. Download PDFs of all research papers.

2. Make sure Model is GPT-4o

3. Set ChatGPT settings: Turn off "Reference saved memories" under Personalization -> Turn off "Improve the model for everyone" under Data controls -> Delete all chats under Data controls

4. Clear ChatGPT memory: Manage memories under Personalization

5. Generate PLS: Paste prompt into new chat -> Add a research paper PDF -> Enter -> Paste ChatGPT's response into a word document with this titling construction: “First Author Last Name, Publication Year, Title of Paper” (For example, Mattingly 2023, Harm perceptions of secondhand e-cigarette aerosol).

6. Create a PDF of the Word Doc with the same title.

7. Clear ChatGPT memory: Manage memories under Personalization

8. Repeat Steps 4-7 until PLSs have been generated for all papers.
