# Supplementary material for: Plain Language Summarization of Environmental Health Research Using Generative AI: Community-Engaged Qualitative Study
Source: J Med Internet Res. 2026 May 13;28:e87118. doi: 10.2196/87118 (PMC13216757; doi:10.2196/87118)
Supplement: Multimedia Appendix 2 [file jmir_v28i1e87118_app2.docx]

**Appendix B: Complete PLS Examples from Focus Groups**

**Research Paper:** McGraw, K. E., Riggs, D. W., Rai, S., Navas-Acien, A., Xie, Z., Lorkiewicz, P., Lynch, J., Zafar, N., Krishnasamy, S., Taylor, K. C., Conklin, D. J., DeFilippis, A. P., Srivastava, S., & Bhatnagar, A. (2021). Exposure to volatile organic compounds – acrolein, 1,3-butadiene, and crotonaldehyde – is associated with vascular dysfunction. Environmental Research, 196, 110903. <https://doi.org/10.1016/j.envres.2021.110903>

- **Prompt A (initial draft)** - *"Summarize the research paper in 600 words at an eighth-grade reading level. The entire summary should be in narrative format."*

This research study explored how exposure to certain air pollutants called **volatile organic compounds (VOCs)**—specifically acrolein, 1,3-butadiene, and crotonaldehyde—affects heart and blood vessel health. VOCs are chemicals released from vehicle exhaust, industrial activities, tobacco smoke, and even household products. While scientists have long known that tiny particles in the air (known as PM2.5) can cause heart disease, less is known about how gaseous pollutants like VOCs might also damage the cardiovascular system. The researchers wanted to find out whether these chemicals contribute to **vascular dysfunction**, which refers to problems with blood vessel function that can lead to high blood pressure and heart disease.

The study analyzed data from **346 nonsmokers** participating in the Louisville Healthy Heart Study, a project examining how pollution influences heart health in Louisville, Kentucky. Participants represented a diverse, urban population with varying levels of cardiovascular risk. To measure exposure to VOCs, the researchers tested each person’s urine for **biological markers (metabolites)** that indicate how much of these chemicals entered their body. They focused on specific urinary metabolites related to acrolein (3HPMA and CEMA), 1,3-butadiene (DHBMA and MHBMA3), and crotonaldehyde (HPMMA).

At the same time, the researchers measured participants’ **blood pressure**, **endothelial function** (how well blood vessels dilate), and levels of **catecholamines**—hormones like epinephrine (adrenaline) and norepinephrine that regulate stress and heart rate. Using statistical models, they examined how the levels of different VOC metabolites related to these measures of heart health. They also used a complex technique called **Bayesian Kernel Machine Regression (BKMR)** to evaluate the combined effects of multiple VOC exposures at once.

The results showed that exposure to **acrolein** and **1,3-butadiene** was strongly linked to poorer blood vessel health. For every increase in urinary 3HPMA (a marker of acrolein exposure), systolic blood pressure rose by about **1 mmHg**. Higher levels of both 3HPMA and DHBMA (a 1,3-butadiene metabolite) were also linked to worse endothelial function, meaning that the blood vessels were less able to relax and widen properly. These findings suggest that even low-level exposure to these VOCs can contribute to **elevated blood pressure and early signs of cardiovascular disease**.

Interestingly, the study found **racial differences** in vulnerability. Black participants showed stronger associations between VOC exposure and blood pressure increases—even though their urinary VOC levels were generally lower than those of White participants. This suggests that social, biological, or environmental factors may heighten the cardiovascular impact of pollution among certain populations. These differences align with broader public health data showing that Black communities in the U.S. often face disproportionate environmental risks and higher rates of heart disease.

The researchers also examined whether stress hormones mediated the relationship between VOC exposure and blood pressure. Although some VOCs were linked to higher levels of catecholamines, the study did not find that these hormones directly explained the rise in blood pressure. However, the data hinted that they might play a modifying role, influencing how the body responds to pollution.

Overall, the findings support the idea that **VOCs are significant but under-recognized contributors to cardiovascular disease risk**. The acrolein metabolite 3HPMA appeared to be the most important driver of elevated blood pressure, suggesting that acrolein may be particularly harmful among the many VOCs people are exposed to daily. This study is one of the first to use biomonitoring data—measuring pollutants inside people’s bodies rather than just in the air—to link VOC exposure with cardiovascular dysfunction in nonsmokers.

The authors note that these results are especially important for **environmental justice** because VOC exposure often comes from industrial sources and vehicle emissions concentrated in lower-income or minority neighborhoods. Reducing exposure to these pollutants could improve cardiovascular health outcomes, especially in vulnerable communities. The researchers conclude that further studies are needed to confirm these findings in larger populations and to explore interventions that can reduce VOC exposure and protect public health.

- **Prompt B (initial draft)** – *"Summarize the research paper in 300 words at a sixth-grade reading level. The entire summary should be in bullet format."*
- **Purpose of the study:**
  The researchers wanted to learn if breathing in certain air pollutants called volatile organic compounds (VOCs) can harm the heart and blood vessels. They studied three specific VOCs: acrolein, 1,3-butadiene, and crotonaldehyde.
- **Why it matters:**
  These chemicals are found in car exhaust, tobacco smoke, and industrial pollution. Over time, they may cause high blood pressure and heart disease, especially for people living near busy roads or factories.
- **Who was studied:**
  The team looked at 346 adults in Louisville, Kentucky, who did not smoke. The group included both Black and White participants.
- **How exposure was measured:**
  Scientists tested urine samples to find signs of VOCs, called metabolites. These show how much of each chemical had entered a person’s body.
- **Heart health tests:**
  Researchers measured blood pressure, how well blood vessels relaxed and widened (called endothelial function), and stress hormones that affect the heart.
- **Key findings:**
  - People with higher levels of acrolein and 1,3-butadiene in their urine had higher blood pressure.
  - These chemicals were also linked to worse blood vessel function, meaning the vessels were stiffer and less healthy.
  - Crotonaldehyde exposure showed weaker links but still suggested possible harm.
  - Black participants showed stronger effects from these chemicals, even though their exposure levels were lower.
- **What this means:**
  Breathing in VOCs can raise blood pressure and damage blood vessels, which can lead to heart disease.
- **Why it matters for health and equity:**
  These pollutants are more common in neighborhoods near highways or factories—places where many lower-income or minority families live. Reducing VOC exposure could help prevent heart disease in these communities.
- **Prompt C (refined draft)** - "*Summarize the provided text using simple and clear language. Start with key findings and then translations for technical terms. Organize the summary using headers for easy reading*.
  - *Include:*
- *Complete title and all authors*
- *Key Findings: List the main results or conclusions here.*
- *Definitions: Translate any technical terms used in the summary.*
- *Introduction: Provide a brief overview of the topic.*
- *Main Content: Discuss the significant points, organized with headers as needed.*
- *Conclusion: Summarize the overall findings and their implications.*
- *Ensure the summary is suitable for a non-expert audience. Do not include methods or results sections in the summary*."

**Complete Title and Authors:**
McGraw, K. E., Riggs, D. W., Rai, S., Navas-Acien, A., Xie, Z., Lorkiewicz, P., Lynch, J., Zafar, N., Krishnasamy, S., Taylor, K. C., Conklin, D. J., DeFilippis, A. P., Srivastava, S., & Bhatnagar, A. (2021). *Exposure to volatile organic compounds – acrolein, 1,3-butadiene, and crotonaldehyde – is associated with vascular dysfunction.* *Environmental Research, 196*, 110903. <https://doi.org/10.1016/j.envres.2021.110903>

**Key Findings**

- People exposed to higher levels of *volatile organic compounds (VOCs)*—especially **acrolein** and **1,3-butadiene**—had higher blood pressure and less flexible blood vessels.
- These chemicals can harm the heart and blood vessels even at low exposure levels.
- Black participants experienced stronger effects from these pollutants compared to White participants, despite having lower exposure levels.
- This study suggests VOC exposure is an overlooked factor in **heart disease risk**, particularly for communities near pollution sources like highways and factories.

**Definitions**

- **Volatile Organic Compounds (VOCs):** Gases released from cars, factories, cigarette smoke, and some household products.
- **Endothelial Function:** How well blood vessels expand and contract to keep blood flowing normally.
- **Metabolites:** Chemicals in urine that show what pollutants have entered the body.
- **Acrolein, 1,3-Butadiene, Crotonaldehyde:** Specific harmful VOCs linked to heart and blood vessel damage.

**Introduction**

This study looked at how everyday exposure to harmful gases called volatile organic compounds (VOCs) affects heart and blood vessel health. These chemicals come from car exhaust, tobacco smoke, and industrial pollution. Researchers wanted to see if people with higher VOC exposure showed early signs of heart problems, such as high blood pressure or damaged blood vessels.

**Main Content**

Scientists studied **346 adults in Louisville, Kentucky**, who did not smoke. They measured VOC exposure by testing urine samples for chemical markers that show how much of each compound had entered the body.

They also measured blood pressure and how well the blood vessels functioned. Using advanced statistical models, the researchers found that higher exposure to **acrolein** and **1,3-butadiene** was linked to worse blood vessel health and higher blood pressure.

Importantly, the study found racial differences in these effects—**Black participants** showed stronger negative responses even at lower exposure levels, suggesting that social and environmental stressors may worsen the impact of pollution.

**Conclusion**

The study concludes that everyday exposure to volatile organic compounds can raise blood pressure and damage the blood vessels, increasing the risk for heart disease. Because VOCs are more common in neighborhoods near factories and highways, this research highlights an **environmental justice concern**. Reducing VOC emissions and improving air quality could help prevent heart disease, especially in vulnerable communities.

- **Final Prompt (incorporating community feedback):** *Summarize using simple, community-accessible language (6th–8th grade). Include citation, definitions, key findings, introduction, and conclusion. Highlight implications for affected communities and environmental justice.*

**Complete Citation (APA format):**
McGraw, K. E., Riggs, D. W., Rai, S., Navas-Acien, A., Xie, Z., Lorkiewicz, P., Lynch, J., Zafar, N., Krishnasamy, S., Taylor, K. C., Conklin, D. J., DeFilippis, A. P., Srivastava, S., & Bhatnagar, A. (2021). *Exposure to volatile organic compounds – acrolein, 1,3-butadiene, and crotonaldehyde – is associated with vascular dysfunction.* *Environmental Research, 196*, 110903. <https://doi.org/10.1016/j.envres.2021.110903>

**Definitions**

- **Volatile Organic Compounds (VOCs):** Harmful gases released from car exhaust, industrial pollution, tobacco smoke, and some home products.
- **Endothelial Function:** How well blood vessels expand and contract to help control blood pressure.
- **Metabolites:** Substances found in urine that show how much of a chemical has entered the body.
- **Environmental Justice:** The fair treatment and involvement of all people in protecting their communities from pollution and environmental harm.

**Key Findings**

- Breathing in VOCs—especially **acrolein** and **1,3-butadiene**—can raise blood pressure and make blood vessels stiffer and less healthy.
- These harmful effects were stronger among **Black participants**, even though their exposure levels were lower than White participants.
- Everyday exposure to these chemicals, even at low levels, can increase the risk of heart disease.
- VOC exposure is higher in communities close to highways, factories, or industrial areas, highlighting a major environmental justice issue.

**Introduction**

This study looked at how breathing in common air pollutants called *volatile organic compounds* affects heart and blood vessel health. These pollutants come from car exhaust, industrial emissions, and cigarette smoke. While most research has focused on tiny particles in the air, this study focused on harmful gases that are harder to see or smell. Researchers wanted to understand how these gases affect people’s health—especially those living in areas with high air pollution.

**Main Content**

The study included **346 adults in Louisville, Kentucky**, who did not smoke. The researchers tested their urine to measure levels of VOCs in their bodies and examined their blood pressure and blood vessel function. They found that people with higher levels of **acrolein** and **1,3-butadiene** in their bodies had higher blood pressure and less flexible blood vessels. These changes are early warning signs of heart disease.

The study also revealed racial differences. **Black participants** were more likely to experience the harmful effects of these pollutants, even when their exposure levels were lower. This finding suggests that pollution affects some groups more severely, possibly due to stress, housing, or unequal environmental conditions.

**Conclusion and Community Implications**

This research shows that **air pollution harms heart health**, even for people who do not smoke. The findings highlight the need for cleaner air and stronger protections for communities exposed to high pollution levels. Since many affected areas are lower-income or majority-Black neighborhoods, improving air quality is not just a health issue—it is a **matter of fairness and justice**.

Reducing VOC pollution from vehicles, industries, and urban areas can lower heart disease risk and help create healthier, more equitable communities. Public awareness, better policies, and community involvement are key to protecting everyone’s right to breathe clean air.
