# Supplementary material for: Plain Language Summarization of Environmental Health Research Using Generative AI: Community-Engaged Qualitative Study
Source: J Med Internet Res. 2026 May 13;28:e87118. doi: 10.2196/87118 (PMC13216757; doi:10.2196/87118)
Supplement: Multimedia Appendix 3 [file jmir_v28i1e87118_app3.docx]

**Appendix C: Feedback Categorization for Summaries**

| Category | Feedback Notes |
| --- | --- |
| Language & Jargon Simplified | "If I see a lot of acronyms and chemical names, I stop paying attention. I would rather have something that can explain it like a good teacher would, even if the original was complicated." "The technical jargon is one of biggest problems. Before you explain the study, I think it would be great to have the meaning of terms that keep showing up." "If a paper you make uses the same jargon as the original scientific paper, then it is not really a summary for us." "I would like it if I felt I could share with my parents and cousins and that they would understand it." |
| Clear Structure & Navigation | "Headers would help me a lot. When everything is in one block of text, I do not know where to look for the main point." "I do not need every small detail from the paper. I want to see the important points organized in a way that makes sense." "I like when information is broken into sections like procedures, data, and conclusion. That makes it easier to go back and find what I need." |
| Identify Key Findings | "Sometimes I can read every sentence and still not know what the researchers found. I want the important stuff pulled out clearly." "I do not need every small detail from the paper. I want to see the important points organized in a way that makes sense." "It would help if the version you made clearly separated what the experiment was about from what it means." |
| Provide Background & Context | "You should have a short introduction that tells me what this is about and why it matters before getting into details." "I do not want the writer to assume I already know the topic. The paper should give me some background." |
| Explain the Implications | "At the end, I want someone to tell me what it all adds up to. What does this mean in real life here in Louisville, and why should I care?" "I need the end to say not just what happened, but what the results might mean for people who live in my neighborhood." |
| Trust and Sources | "I like knowing exactly what source the summary came from. If you include the full details of the paper, I might be more likely to trust it and look into it myself later." |
| Variable Detail Level | "Sometimes I just want the bottom line, but other times I want a little more explanation -- so give me both." |
| Explanatory Narrative | "It should walk me through the main content step by step, not just tell me what to think." |
